# miR-20b is up-regulated in brain metastases from primary breast cancers

**DOI:** 10.18632/oncotarget.3664

**Published:** 2015-03-26

**Authors:** Aamir Ahmad, Kevin R. Ginnebaugh, Seema Sethi, Wei Chen, Rouba Ali, Sandeep Mittal, Fazlul H. Sarkar

**Affiliations:** ^1^ Department of Pathology, Wayne State University School of Medicine and Karmanos Cancer Institute, Detroit, Michigan, USA; ^2^ Department of Oncology, Wayne State University School of Medicine and Karmanos Cancer Institute, Detroit, Michigan, USA; ^3^ Department of Neurosurgery, Wayne State University School of Medicine and Karmanos Cancer Institute, Detroit, Michigan, USA

**Keywords:** miR-20b, breast cancer, brain metastasis

## Abstract

Brain metastases are frequent in patients with advanced breast cancer and are associated with poor prognosis. However, unique molecular biomarkers have not yet been established. We hypothesized that microRNA-20b (miR-20b) plays a role in breast cancer brain metastasis. Our study cohort comprised of eleven breast cancer patients with brain metastasis and nine control patients (age, stage, and follow-up matched) with breast cancer without brain metastasis. Cases were reviewed microscopically to select tumor blocks with >50% tumor cells, RNA was extracted from formalin-fixed paraffin-embedded (FFPE) tumor tissue blocks and expression of miR-20b analyzed using qRT-PCR. We further tested the effect of miR-20b overexpression on colony formation and invasion *in vitro* using MCF-7 and MDA-MB-231 cells. In the patient-derived samples, miR-20b expression was significantly higher in brain metastases of breast cancer patients, compared to primary breast tumors as well as the patients without brain metastasis. miR-20b also significantly induced the colony formation and invasiveness of breast cancer cells. Further, miR-20b levels were observed to be high in brain-metastasizing cells, compared to bone-metastasizing cells. Together, our findings suggest a novel role of miR-20b in breast cancer brain metastasis that warrants further investigation for its potential to be developed as prognostic and/or therapeutic target.

## INTRODUCTION

Breast cancer is one of the most commonly diagnosed solid malignancies and the leading cause of cancer-related deaths among women. In 2015, breast cancer alone is expected to account for 29% of all new cancers among women in the United States [1]. Medical advancements in recent years, combined with aggressive screening programs, have significantly reduced breast cancer-related mortality, but the numbers are still too high. Among millions of Americans with a history of cancer, breast cancer tops the list of most prevalent cancers among women with 41% [2]. Breast cancer is a complex disease with many subtypes and one primary reason that makes breast cancer particularly lethal is its metastasis to distant organs. Breast cancer is the second leading cause of brain metastases and significantly impacts patient's quality of life [3].

Development of effective systemic treatments of primary breast cancers has resulted in higher incidence as well as mortality associated with metastatic disease. One of the biggest clinical challenges for the effective management of brain metastatic breast cancers is the lack of validated molecular targets [4]. In recent years, microRNAs (miRNAs) have emerged as promising prognostic and therapeutic targets for metastatic breast cancers [5-8]. miRNAs have also been implicated in the brain metastases of breast cancers and the ones investigated in this context include miR-7 [9], miR-1258 [10] and miR-146a [11]. Recently, we reported [12] elevated miR-10b levels in primary breast cancer specimens of patients who subsequently developed brain metastasis, compared to those who did not. miR-20b is another microRNA that has been shown to be involved in breast tumorigenesis [13, 14] and, therefore, this study was undertaken to determine the role, if any, of miR-20b in breast cancer brain metastasis.

The oncogenic action of miR-20b in breast cancer models has been suggested to involve suppression of phosphatase and tensin homolog (PTEN) [13, 14] and BRCA1 (breast cancer 1, early onset) [13]. Interestingly, both of these tumor suppressors, PTEN [15] and BRCA1 [16], have been linked to brain metastasis of primary breast cancers, thus providing an indirect link between miR-20b and brain metastatic breast cancers. In the present study, we investigated the endogenous expression levels of miR-20b in breast cancer patients-derived primary tumor specimens as a pilot study. We compared the levels of miR-20b in brain metastatic breast cancers to the levels in breast cancer patients without brain metastasis.

## RESULTS

### Patients

**Figure 1:**
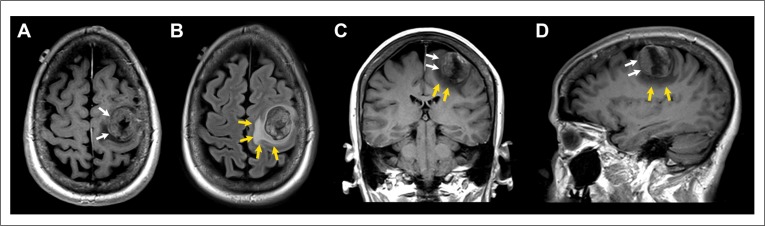
Patient with solitary breast cancer brain metastasis involving the posterior aspect of the left middle frontal gyrus Post-contrast T1-weighted axial (**A**), coronal (**C**), and sagittal (**D**) images and fluid-attenuated inversion recovery (FLAIR) axial image (**B**) showing a 2.5 × 3.3 × 3.1 cm mass (white arrows) with extensive peritumoral vasogenic edema (yellow arrows). Of note, this patient had the highest expression of miR-20b in the resected tumor specimen.

A total of 20 breast cancer cases met the study criteria. These included 11 breast cancer cases with brain metastasis and 9 age, stage and follow-up matched breast cancer cases without brain metastasis [12]. Figure 1 shows the typical radiographic features of a patient who underwent resection of a solitary breast cancer brain metastasis.

### Effect of miR-20b on invasion and colony formation of breast cancer cells

**Figure 2:**
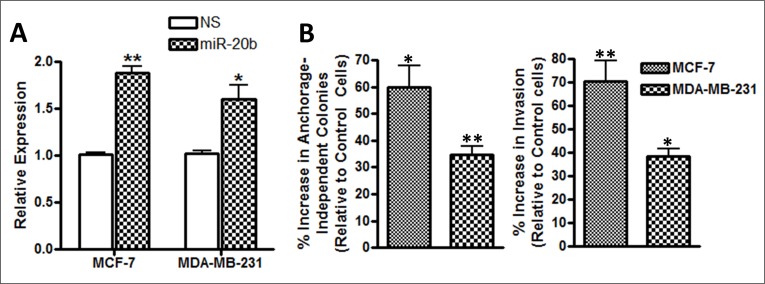
(**A**) MCF-7 and MDA-MB-231 breast cancer cells were transfected with non-specific pre-miRNA controls (NS) or pre-miR-20b (miR-20b). Relative expression of miR-20b was determined by quantitative RT-PCR. RNU48 was used as internal miRNA control against which the data was normalized. (**B**) miR-20b-transfected MCF-7 and MDA-MB-231 cells exhibited significantly increased anchorage-independent colony formation in soft agar, and invasiveness, compared to control cells (non-specific pre-miR-transfected cells). *p < 0.05, **p < 0.01.

There is evidence to suggest that miR-20b is involved in regulation of estrogen receptor (ER) [17], so, we chose ER-positive MCF-7 cells as our model to investigate *in vitro* effects of miR-20b modulation. Additionally, triple-negative breast cancers (TNBC) are linked to brain metastases and, therefore, we chose a TNBC cell line, MDA-MB-231, as our other model. We transfected cells with pre-miR-20b oligonucleotides and observed the effects on anchorage-dependent colony formation as well as invasion. Pre-miR-20b transfections results in increased expression of miR-20b, suggesting efficient transfections (Figure 2A). We tested colony formation by these cells under anchorage-independent conditions in soft agar and observed 59.9% more colonies in pre-miR-20b-transfected MCF-7 cells (p=0.0233) and 34.4% more colonies in pre-miR-20b-transfected MDA-MB-231 cells (p=0.0040) (Figure 2B). When tested for invasion, miR-20b transfected cells were significantly more invasive. Relative to control cells, we observed 70.3% increase in invasiveness of MCF-7 cells (p=0.0003) and 38.5% increase in invasiveness of MDA-MB-231 cells (p=0.0186) that were transfected with pre-miR-20b (Figure 2B).

### miR-20b in patients with brain metastases

**Figure 3:**
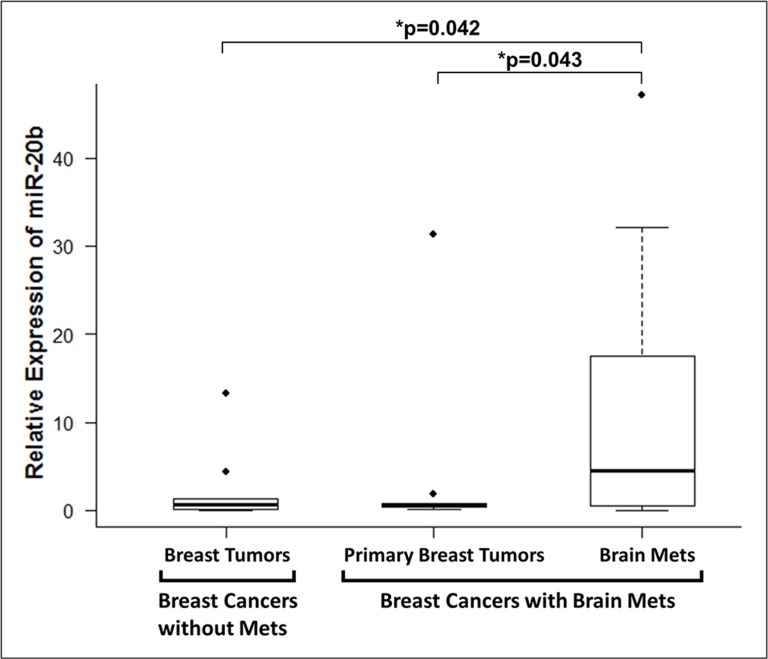
Relative expression of miR-20b in patient samples miRNAs extracted from FFPE samples of breast cancer patients with or without breast cancer brain, and analyzed for expression of miR-20b. RNU48 was used as miRNA control against which the data was normalized. *p < 0.05.

**Figure 4:**
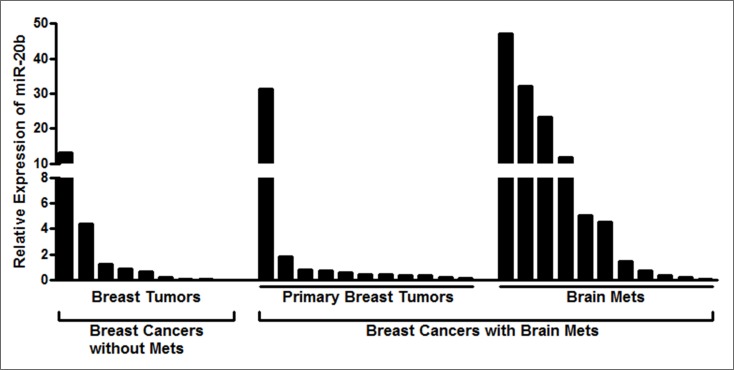
Relative expression of miR-20b in individual patient samples miRNAs extracted from FFPE samples of individual breast cancer patients with or without breast cancer brain metastasis were analyzed for expression of miR-20b. RNU48 was used as miRNA control against which the data was normalized.

Next, we measured the levels of miR-20b in breast cancer patients without metastases vs. those with brain metastasis. For the patients with brain metastasis, we evaluated the levels of miR-20b in the primary breast tumor as well as the brain metastasis of the same patient. miR-20b levels were significantly higher in the brain tumor samples of the patients with brain metastases, compared to the samples from patients without brain metastases (Figure 3). Data was analyzed using the one-sided two-sample t test on log-transformed data and observed to be statistically significant (p=0.042). The levels of miR-20b in brain metastases were also found to be significantly higher than the primary breast tumor samples of the same patients (p-0.043), as analyzed by one-sided one-sample paired t test on log-transformed data (Figure 3). We further analyzed the expression levels of miR-20b in individual patients' samples, and compared miR-20b levels in 9 breast cancer patients without brain metastasis with the levels in primary breast tumors and brain tumors of 11 breast cancer patients with brain metastases. As shown in Figure 4, brain metastasis of breast cancer patients exhibited higher levels of miR-20b, with some patient-specific differences, as expected in such studies. With the exception of two patients, miR-20b levels were observed to be low in breast cancers without metastasis. When compared within the samples from breast cancer patients with brain metastasis, primary breast tumors had low levels of miR-20b with just one patient sample showing very high level while the brain tumors of more than half of the patients had significantly increased levels of miR-20b.

### miR-20b in brain vs. bone-seeking cells

**Figure 5:**
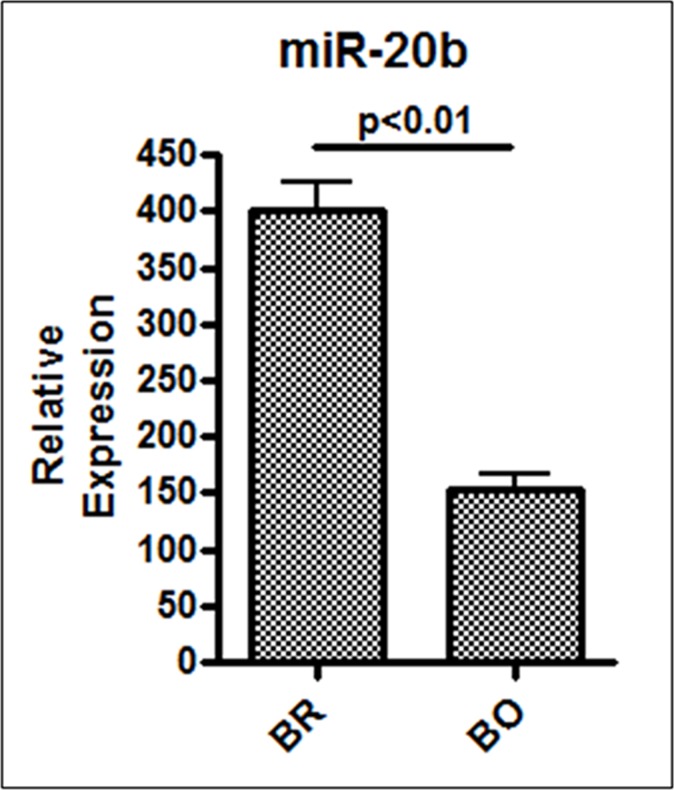
Relative expression of miR-20b in brain vs. bone-seeking breast cancer cells Expression of miR-20b was determined by quantitative RT-PCR. RNU48 was used as internal miRNA control against which the data was normalized. *BR, Brain-metastasizing MDA-MB-231 cells; BO, Bone-metastasizing MDA-MB-231 cells*. *p < 0.01.

The results from patient-derived samples suggested an involvement of miR-20b in brain metastasis of breast cancer. In order to test whether expression of miR-20b is a specific predictor of brain metastasis, we checked its expression in brain vs. bone-seeking derivatives of MDA-MB-231 cells. These brain- and bone-seeking cells were established by repeated sequential passages in nude mice and *in vitro* of metastatic cells obtained from brain and bone metastases, respectively [18]. When analyzed for endogenous miR-20b levels, we observed greater than 2.5-fold increased levels of miR-20b in brain-seeking cells, compared to bone seeking cells (p < 0.01) (Figure 5). Significantly higher expression of miR-20b in brain-seeking cells is indicative of the role of miR-20b in brain-specific metastasis of breast cancer cells. These results suggest that miR-20b might be a specific biomarker for breast cancers with high propensity to metastasize to brain.

## DISCUSSION

The risk factors for breast cancer brain metastasis, which occur in up to 30% of patients with advanced disease [19, 20], are not well known. It has been proposed that overexpression/amplification of HER2/neu (human epidermal growth factor-2) might pose a particular risk with an estimated 30-50% of advanced HER2-positive breast cancers progressing to brain metastases [21]. Another subtype of breast cancer with propensity of brain metastasis is the TNBC. In a retrospective study on breast cancer patients with brain metastases [22], the median time from first recurrence to brain metastasis was determined to be 5.5 months for TNBC patients as compared to 9.6 months for HER2-overexpressing patients. In another retrospective study [23], the overall survival of brain metastatic breast cancers was lower in TNBC as compared to HER2 overexpressing breast cancer patients (4.9 months vs. 11.5 months, respectively). It appears that a higher proportion of HER2 overexpressing breast cancers metastasize to brain but the TNBCs that metastasize to brain are relatively more aggressive. Our knowledge on these correlations is still evolving and it is not well established how breast cancer subtypes are associated with brain metastasis. HER2-overexpressing breast cancers present with an opportunity for targeted intervention. On the contrary, TNBCs have no validated therapeutic targets and are more challenging to treat. Even with approved therapies against HER2-overexpressing breast cancers, the results have not been very encouraging because most of the agents cannot effectively cross the blood-brain barrier [24].

The association of breast cancer subtypes with risk of brain metastasis has been the focus of many studies because this might help identify patients who can be intervened early to possibly eliminate chances of developing brain metastatic disease. A bigger challenge is to validate molecular markers that can be exploited for targeted therapies. In recent years, miRNAs have been proposed as regulators of breast cancer metastases [7] and there is evidence that some of the miRNAs, namely miR-7 [9], miR-10b [12], miR-146a [11] and miR-1258 [10] play a role in brain metastases of breast cancers. It is interesting to note that a majority of these miRNA are tumor suppressive and are down-regulated in brain metastasis. For example, miR-7 was found to be down-regulated and its target KLF4 up-regulated in brain metastases [9]. KLF4 is associated with cancer stem cell (CSC) phenotype and it up-regulation might suggest an involvement of CSCs in brain metastasis of breast cancers. The brain metastases suppressing action of miR-146a was proposed to involve up-regulation of its target heterogeneous nuclear ribonucleoprotein C1/C2 (hnRNPC) and the resulting up-regulation of factors such as MMP-1, uPA and uPAR [11]. miR-1258 regulated brain metastasis through suppression of heparanase [10] and cells stably transfected with miR-1258 had lowered levels of heparanase with reduced metastases. Our recent work focused on miR-10b [12] revealed a unique association of this miRNA with brain metastasis in patient-derived samples. miR-10b was particularly high in breast cancers with brain metastasis, compared to breast cancers that did not metastasize. We now report a similar function of miR-20b. The results described here point to higher overall levels of miR-20b in brain metastasis, compared to breast cancers without metastasis.

A number of reports have evaluated the role of miR-20b in human cancers [25, 26] and like many other miRNAs, the results are conflicting. Some studies found a down-regulated miR-20b supporting its tumor suppressive function [27-29] while others reported elevated levels supporting an oncogenic role [13, 14, 30-33]. Our results are indicative of an oncogenic role because we found elevated levels of miR-20b in brain metastases. In an earlier report [9], it was noted that the down-regulation of miR-7 along with up-regulation of its target KLF4, was inversely associated with brain metastasis-free survival but was not associated with bone metastasis. These results suggested that modulated miRNA expressions might determine the specific metastasis of breast cancer cells to brain. We observed an elevated miR-20b in brain-seeking cells compared to bone-seeking cells. It thus appears that even miR-20b might play a role in brain-specific metastasis of breast cancer cells, an idea that needs further investigation. Metastatic breast cancer is lethal, irrespective of the final organ of metastasis, but the published results with miR-7 and our current results point to the possibility of a miRNA signature that can help predict the elevated risk of brain metastases.

A number of studies have identified PTEN to be a target of miR-20b [13, 32, 33]. Such validation includes studies conducted in breast cancer models [13, 14]. PTEN is a tumor suppressor that is down-regulated in human cancers. A survey of literature reveals a role of PTEN in brain metastasis of human cancers of different origins. In a study [15] that used array-comparative genomic hybridization and microsatellite analysis to compare patterns of chromosomal aberrations in primary tumors vs. brain metastases from breast cancer patients, PTEN was significantly down-regulated in brain metastases compared with non-metastatic primary tumors. A similar role of PTEN was later observed in brain metastases of non-small cell lung cancers [34]. Loss of PTEN was also reported to correlate significantly with shortened time to brain metastases in melanoma patients [35]. It thus appears that PTEN loss is relevant to brain metastases from cancers of different primary origins, and a regulatory role of miR-20b, as suggested by our present results, will be interesting to evaluate in future studies.

In conclusion, a significant number of advanced breast cancers metastasize to brain, with molecular biomarkers being a subject of ongoing investigations. Our results suggest that miR-20b can be a candidate biomarker for the identification of patients at high risk of brain metastatic disease. Combined with our earlier published results [12] and those from other groups [9-11], it is possible that brain metastatic breast cancer cells have a distinct signature. In this burgeoning era of personalized medicine, finding such molecular signature would represent a significant step forward towards the ultimate goal of optimizing targeted therapies as well as designing novel approaches to prevent, delay and eliminate brain metastatic disease.

## MATERIALS AND METHODS

### Breast cancer patients

A retrospective search was done through the computerized database at the Department of Pathology, Karmanos Cancer Institute, Wayne State University, for breast cancer cases with brain metastasis, as described previously [12]. The period of analysis was cases diagnosed between January 1994 and December 2011. The study was approved by the Institutional Review Board.

### Tumor selection

The histopathological glass slides were microscopically reviewed to select the tumor block with preserved, viable tumor tissue comprising over 50% of tumors in the paraffin block, and tumor area was marked. Slides with large areas of necrosis were excluded from the study. Ten sections of 10 μM thickness were cut from each selected block marked for tumor areas and avoiding normal tissues [12].

### Cell lines

Human breast cancer cell lines MCF-7 and MDA-MB-231 were cultured in DMEM media with 10% fetal bovine serum and 1% penicillin/streptomycin. Brain and bone-specific derivatives of MDA-MB-231 cells were obtained from Dr. Yoneda's lab [18]. All cells were cultured in 5% CO_2_–humidified atmosphere at 37°C. The cell lines have been tested and authenticated in core facility (Applied Genomics Technology Center at Wayne State University) by short tandem repeat profiling using the PowerPlex 16 System from Promega.

### RNA extraction and Real-Time RT-PCR

RNA was extracted from the FFPE tumor samples using the Qiagen kits, as per the manufacturers' protocol. Expression of miR-20b was assessed using quantitative RT-PCR, as described previously [12]. For the cultured cells, total RNA was isolated using mirVana miRNA isolation kit (Ambion). The miRNA levels were determined using miRNA-specific Taqman MGB probes from the Taqman MicroRNA Assay (Applied Biosystems). The relative amounts of miRNA were normalized to the endogenous control gene RNU48.

### miR-20b transfections

Cells were seeded at 2.5 × 10^5^ cells per well in six-well plates and transfected with pre-miR-20b or pre-miRNA-negative controls (Ambion) at a final concentration of 200 nM using DharmaFECT1 transfection reagent (Dharmacon). After 2 days of transfection, cells were passaged and transfected twice again before using these cells for invasion assays.

### Anchorage-independent (soft agar) colonization assays

3 × 10^4^ cells were plated in 0.5 ml of culture medium containing 0.3% (w/v) top agar layered over a basal layer of 0.7% (w/v) agar (with culture medium and the supplements) in 24-well plates. After appropriate culture time (3 weeks for MDA-MB-231 and 4 weeks for MCF-7 cells), colonies (>50 cells) were counted. Experiments were carried out in triplicate, and results presented are representative of two independent observations.

### Cell invasion assay

Cell invasion assays were performed using 24-well Transwell Permeable Supports with 8 μM pores (Corning, Lowell, MA). Cells (control and pre-miR-20b-transfected) were suspended in serum free medium and seeded into the Transwell inserts coated with growth factor reduced Matrigel (BD Biosciences, Bedford, MA). Bottom wells were filled with media containing complete media. After 24 h, cells were stained with 4 μg/ml calcein AM (Invitrogen) in PBS at 37°C for 1 h and detached from inserts by trypsinization and fluorescence of the invaded cells was read in ULTRA Multifunctional Microplate Reader (TECAN, San Jose, CA).

### Data analysis

The data are presented as the mean values ± SD. A p value < 0.05 was considered to be statistically significant. To compare the miR-20b in tissues from breast cancers with or without brain metastasis, one sided two sample t test on log-transformed data was used. To compare miR-20b in tissues from paired primary breast tissues and brain metastases, one sided one sample paired t test on log-transformed data was used. All p values were raw and not adjusted for multiple testing. The statistical analyses for human tissue samples were performed with software R. Two sided two sample t test was used for the rest of analyses (using PRISM).
